# Measuring Gambling Outcome Expectancies in Adolescents: Testing the Psychometric Properties of a Modified Version of the *Gambling Expectancy Questionnaire*

**DOI:** 10.1007/s10899-021-10053-y

**Published:** 2021-07-07

**Authors:** Maria Anna Donati, Jeffrey L. Derevensky, Beatrice Cipollini, Laura Di Leonardo, Giuseppe Iraci Sareri, Caterina Primi

**Affiliations:** 1 Department of Neuroscience, Psychology, Drug and Child’s Health, Section of Psychology, University of Florence, via di San Salvi 12- Padiglione 26, 50135 Florence, Italy; 2 Department of Education and Counselling Psychology, McGill University, Montreal, Canada; 3 Gruppo Incontro, Pistoia and CEART (Coordinamento Enti Ausiliari Regione Toscana), Pistoia, Italy

**Keywords:** Adolescents, Gambling, Outcome expectancies, *Gambling Expectancy Questionnaire—Modified*, Invariance

## Abstract

The *Gambling Expectancy Questionnaire* (GEQ; Gillespie et al. 2007a) is a 23-item scale assessing three positive outcome expectancies (*Enjoyment/Arousal*, *Money*, *Self-Enhancement*) and two negative outcome expectancies (*Over-Involvement, Emotional Impact*) related to gambling. It is the most used instrument to assess gambling outcome expectancies in adolescents and it has good psychometric properties. To allow a greater and more useful application of the scale, the present study aimed to modify the GEQ to make it usable with all adolescents, regardless of their gambling behaviour and to verify its psychometric properties. To that aim, the items were modified and the response scale was reduced from a seven-point to a five-point Likert scale. To verify the adequacy of the modified scale, two studies were conducted among Italian adolescents. In the first study (n = 501, 75% males, Mage = 16.74, *SD* = .88), after having removed four items and relocating another through explorative factor analysis, the original five-factor structure of the scale was confirmed by applying a confirmatory factor analysis. Reliability and validity evidence were also provided. The second study (n = 1894, 61% males, Mage = 15.68, *SD* = .71) attested its invariance across gambling behaviour status and gender. The modified version of the GEQ (GEQ – MOD) can be profitably used for research and preventive purposes with youth.

## Introduction

International studies report that up to 80–99% of adolescents engage in some forms of gambling (e.g., Splevins et al. 2010) and that between 0.2 and 12.3% meet criteria for pathological gambling behavior (Calado et al. 2017). Given the harms related to gambling behavior for adolescents, several studies have been conducted to identify risk factors for problem gambling (Dowling et al. 2020). Consistent with the expectancy theory, that posits that the choice to engage in a given behaviour is influenced by an individual’s expectations of the reinforcing or punishing effects of engagement in that behaviour (e.g., Aarons et al. 2001; Fromme et al. 2000; Lewis-Esquerre et al. 2005), *gambling outcome expectancies* (GOEs) have been found to be one robust risk-factor for problem gambling in adolescents (e.g., Dowling et al. 2018). *Outcome expectancies* (OEs) are conceptualized as mental “if…then” contingencies (Goldman et al. 1999), representing beliefs about the occurrence of specific outcomes as a result of a particular behaviour (Olson et al. 1996). In detail, they correspond to such a thought: “After engaging in one behaviour, I expect X (Kuntsche et al. 2010). In the gambling field, OEs refer to the anticipated positive/negative outcomes that occur from one’s gambling behaviour (Stewart et al. 2005; Stewart et al. 2014). Research shows that positive OEs (e.g., Emond et al. 2010; Michalczuk et al. 2011; Teeters et al. 2015) and negative OEs (e.g., St-Pierre et al. 2014; Wickwire et al. 2010; Wohl et al. 2006) have a role in predicting problem gambling in young people.

GOEs are socio-culturally influenced. For instance, the observation of parental gambling behaviour contributes to the development of adolescents’ gambling-related expectancies, intended as beliefs about the future outcome of engaging in gambling (Campbell et al. 2010) and OEs fully mediate the relationship between parent-to-offspring problem gambling (Dowling et al. 2018). Due to their socio-cultural nature, GOEs can be present years before the first gambling experience occurs and represents an important determinant of gambling behaviour (e.g. Gillespie et al. 2007b). The spread of gambling behaviours among youth and the pervasiveness of gambling advertisements may also normalize gambling behaviour and decrease risk expectancy (Binde et al. 2019; Parke et al. 2015). The expansion of gambling habits and the availability of gambling opportunities may also influence the development of GOEs. As youth are exposed to more and more gambling advertisements, they are more likely to perceive gambling as a normative activity that is both desirable and safe (Monaghan et al. 2008). Once gambling behaviour is established, specific GOEs provide first-hand information leading to the development of stronger expectancies and the confirmation of existing expectancies further reinforces gambling involvement. Thus, a better understanding of GOEs, even before the initiation of gambling behaviour for adolescents, could facilitate the development of more tailored prevention interventions, as those who have never experienced a certain behaviour may have specific OEs (Kuntsche et al. 2007).

To assess GOEs in adolescents, there is a paucity of instruments (Gillespie et al. 2007a; Wickwire et al. 2010; Wong et al. 2012). Among them, the *Gambling Expectancy Questionnaire* (GEQ; Gillespie et al. 2007a) is the most used internationally with Caucasian young people (e.g., Dowling et al. 2018; Gillespie et al. 2007b; St-Pierre et al. 2014). Indeed, with respect to the other existing tools, not only material and social benefits/costs are assessed, as in the *Adolescent Gambling Expectancies Survey* (AGES; Wickwire et al. 2010) and in the *Chinese Adolescent Gambling Expectancy Scale* (CAGES; Wong et al. 2012), but also the positive outcomes related to enjoyment and arousal, i.e., positive affect and self-enhancement, intended as the social acceptance and independence due to gambling habits. In terms of negative GOEs, the scale allows to assess perceived negative outcomes referred to over-involvement and emotional impacts. Moreover, it has been developed through focus groups with adolescents, a method that was revealed to be useful to capture the broadest item content and the terminology employed by adolescents (Kouimtsidis et al. 2014). In psychometric terms, the original scale showed good internal consistency and has been found to discriminate across groups of gamblers, as those who gambled most frequently scored highly on each of the three positive GEQ subscales, with the *Enjoyment/Arousal* domain proving to be the strongest predictor of problem gambling (Gillespie et al. 2007b). Consistent with what was found with other instruments (e.g., Wickwire et al. 2010; Wohl et al. 2006), the negative expectations were found to be positive predictors of problem gambling (Dowling et al. 2018; St-Pierre et al. 2014), consistent with the immediacy assumption theory (Gillespie et al. 2007b; Wickwire et al. 2010) that proposes that the potential for immediate gains of gambling outweigh more distal negative outcomes. Consistently, Gillespie et al. (2007b) found that problem gamblers had higher scores in the *Over-Involvement* subscale compared to at-risk and social gamblers.

Despite the GEQ’s described advantages, some critical issues can be identified. Specifically, adolescents are asked to indicate the extent to which they expect each of the listed outcomes by referring to their own gambling experience. Consequently, it is not suitable for those adolescents who do not want to gamble and for younger adolescents that have not yet become social/recreational gamblers, although it has been used also with adolescent non-gamblers (Gillespie et al. 2007b). This point represents a great limitation for research – as it does not allow us to understand which GOEs specifically characterize adolescent non-gamblers, while we know that also adolescents who have never experienced a certain behaviour may have positive OEs (Kuntsche et al. 2007) and, specifically, some studies have reported expectancies exist even before gambling initiation (e.g., Gillespie et al. 2007b; Wickwire et al. 2010; Wong et al. 2012). A better understanding of GOEs, even before the initiation of gambling behaviour for adolescents, could facilitate the development of more tailored prevention interventions.

Following these premises, the goal of the current work was to make the GEQ suitable also to adolescent non-gamblers. To that aim, we modified the GEQ items such that adolescents are required to indicate their responses as to what would happen if they were to gamble. In detail, the original introductive statement: “*Some questions will ask you about what you expect to happen when you gamble*”, was changed into: “*If you were gambling, gambling would make you…*”. This statement was modified for the 23 original items, indicating positive and negative OEs, such as “*have fun*” and “*feel more relaxed*”. To better capture the expectancy strength, we also modified the response scale, switching from a seven-point Likert scale – from 1 = *no chance* to 7 = *certain to happen* – to a five-point Likert scale ranging from 1 = *totally disagree* to 5 = *totally agree*, that appears to be less confusing and to increase the response rate (Devlin et al. 1993; Hayes 1992).

In order to test the adequacy of the modified scale, as a first step, the dimensionality, reliability and validity, were investigated (Study 1). Consistent with the original scale (Gillespie et al. 2007a), we aimed at confirming a five-factor solution by conducting exploratory factor analysis (EFA) and confirmatory factor analysis (CFA). Reliability was also analysed by calculating both the Cronbach’s alpha values and the McDonald’s Ω values, given the criticism against the Cronbach coefficient (Deng et al. 2017) and in light of the fact that the omega coefficient is a measure that overcomes the deficiencies of Cronbach’s alpha (McDonald 1999). We hypothesized that we would obtain high internal consistency values, following Gillespie et al. (2007a). Moreover, validity of the scale was investigated by analysing the relationships with gambling frequency and problem gambling severity, aiming at confirming results of previous studies (Dowling et al. 2018; Gillespie et al. 2007b; St-Pierre et al. 2014).

To assess validity of a measurement instrument, measurement invariance should be analyzed. The ability of a measurement tool to function effectively in different groups of respondents is fundamental as it allows us to determine whether the detected differences are related to group membership rather than the construct that is being measured (i.e., whether a measure is biased because people with similar characteristics who belong to different groups provide markedly different answers). Thus, any findings that address group differences in youth relative to GOEs must be interpreted with caution. If observed group differences have been obtained by employing non-invariant scales across those groups, the overall findings might be misleading, as it is impossible to ascertain whether these differences reflect actual differences in gambling-related OEs among the different groups of adolescents, or whether they reflect differences related to group membership.

Nevertheless, an instrument able to equivalently assess GOEs in non-gamblers and gamblers is still missing. Thus, it is not possible to evaluate differences and similarities of expectancies related to gambling across these two relevant groups, which are under attention of an increasing number of studies aimed at understanding the specific protective factors among who do not chose to gamble (e.g., Labrador et al. 2020; Lalande et al. 2013; León-Jariego et al. 2020).

The study of gender differences of GOEs among adolescents similarly represents a critical issue (St-Pierre et al. 2014) as specific expectancies may differ by gender. For instance, among Canadian adolescents, boys reported more positive OEs, while girls were more likely to endorse the negative emotional impact expectancy. Among male youth, a higher endorsement of the monetary gain and enjoyment/arousal items was also significantly associated with both gambling frequency and gambling severity (Gillespie et al. 2007b). Simmons et al. (2016) reported that gender moderates the relationships between OEs and both gambling frequency and gambling severity among African-American adolescents. More specifically, boys expecting positive outcomes from gambling in terms of affect and self-evaluation reported a higher level of problem gambling. Conversely, for girls, only the expectation of positive self-evaluation was related to higher levels of problem gambling. Moreover, the value of GOEs in the prediction of gambling severity may differ for adolescent males and females (Gillespie 2010; Gillespie et al. 2007b). Nevertheless, the documented gender differences in gambling-related OEs have been found without previously proving measurement invariance across genders of the instruments used to assess GOEs. For these reasons, measurement equivalence of the developed scale across non-gamblers and gamblers and boys and girls were investigated (Study 2).

## Study 1

The aim of this study was to analyze the dimensionality, reliability and validity of the GEQ as modified in the introductive statement and response scale. For the original GEQ, Gillespie et al. (2007a) reported a five-factor structure which accounted for 66.8% of the overall variance, with the *Enjoyment/Arousal* factor explaining the highest proportion of variance. Moderate positive correlations were found among the three positive subscales, as well as between the two negative subscales. Significant, positive, albeit small correlations were found between the positive GOEs subscales and *Over-Involvement*, as well as between *Emotional Impact* and *Self-Enhancement*, while small but significant correlations were found between *Enjoyment/Arousal*, *Money* and *Emotional Impact*.

Internal consistency for the revised scale was analysed by calculating both the Cronbach’s alpha values and the McDonald’s Ω values, while validity of the scale was investigated through relationships with gambling frequency and problem gambling severity among adolescents. First, bivariate correlations between the GEQ dimensions and gambling frequency and gambling problem severity, respectively, were computed. Then, we conducted multiple linear regression analyses in order to identify the dimensions – and their specific weight – related to the two measures of gambling behavior.

## Methods

### Participants

The participants consisted of 501 Italian adolescents (75% males) between the ages of 14 and 20 (*M* = 16.74, *SD* = 0.88). The sample was recruited from different high schools in the centre of Italy (19% vocational school, 45% technical school, 36% lyceum). A study protocol in accordance with the criteria of the Declaration of Helsinki was reviewed and approved by each head teacher and school board of the participating schools. The study protocol presented the goal and methodology of the study explaining that students would complete a research protocol about their gambling habits and perceived expectancies/outcomes. Students received an information sheet that assured them of confidentially and anonymity and provided written informed consent. Parents of minors provided consent on behalf of their children.

### Measures and Procedure

Preliminarily, socio-demographic information was requested. To avoid a predetermined binary vision of adolescents' gender, we left open the question. The variable was made binary only in the data processing phase. Age in terms of years and months was also requested.

The modified version of the 23-item *Gambling Expectancy Questionnaire* (GEQ – MOD) was used to assess gambling outcome expectancies. The GEQ – MOD items were translated into Italian using a forward-translation method. Subsequently, a small group of adolescents completed the modified scale to verify its adequacy in terms of comprehensibility.

To investigate gambling behavior, we administered the *Gambling Behaviour Scale for Adolescents* (GBS-A; Primi et al. 2015). The GBS-A is composed of two sections. The first section consists of unscored items investigating gambling frequency. Ten items assess the frequency (never, sometimes in the year, sometimes in the month, sometimes in the week, daily) of participation during the last year in ten gambling activities (card games, bets on games of personal skill, bets on sports games, bets on horse races, bingo, slot machines, scratch cards, lotteries, online games and private bets with friends). Based on their responses to this section, participants were identified as non-gamblers (no gambling behaviour) or gamblers (gambling on at least one activity; Welte et al. 2009). The second section consists of nine scored items assessing the DSM-5 diagnostic criteria for Gambling Disorder. Each item is evaluated on a three-point Likert scale ranging from 0 (*Never*) to 2 (*Often*). Based on the responses to this section, it is possible to derive an Item Response Theory-based score for each respondent. Following this IRT-based scoring procedure, respondents can be classified into non-problem gamblers, at-risk gamblers and disordered gamblers. The GBS-A has been shown to be unidimensional and useful for mid- to high levels of Gambling Disorder severity (Donati et al. 2017).

The scales were administered by researcher assistants and individually during class time, in this order: GEQ – MOD and then the GBS-A. Students were provided with a brief introduction to the study and with instructions on completing the surveys and were assured confidentiality. Answers were collected in a paper-and-pencil format and data collection was completed in approximately 20–30 min.

## Results

Prior to conducting the analyses, missing values were assessed. Concerning the GEQ – MOD scale, any cases with more than 10% of items (Kline 2010) were reported. For cases with less than 10% of missing values, these were replaced with the subject's mean in that subscale. As for the GBS-A, missing data were not allowed to exceed 10% of the total cases in the sample (Kline 2010). When missing data exceeded 10%, we decided to exclude the case. Only 5 cases were excluded for the first section (gambling frequency). For the second section (gambling problem severity), 13 cases were excluded.

### Adolescent Gambling Behavior

The results indicated that 75% of the respondents (n = 374) have gambled at least once in the past 12 months. The activities that were most frequently engaged in were scratch-cards (52%), bingo (38%) and sports bets (35%). Concerning problem gambling, 81% were non-problem gamblers, 11% were identified as at-risk gamblers and 8% were disordered gamblers.

### Dimensionality of the GEQ – MOD

Univariate distributions of the GEQ – MOD items were examined to assess normality. Skewness and kurtosis indices were between -1 and + 1, except for a few items, which were slightly outside the range of normality for the kurtosis value (Table 1). However, the deviation of a few items from normality could be considered negligible (Ghasemi et al. 2012).

**Table 1:** Means (M), standard deviations (SDs), skewness and kurtosis of the twenty-three items of the GEQ in the modified version

Item	M	SD	Skewness	Kurtosis
1	3.02	1.21	− .15	− .86
2	2.32	1.12	.63	− .30
3	2.86	1.21	− .09	− .94
4	3.08	1.19	− .39	− .85
5	2.62	1.23	.31	− .86
6	2.87	1.19	− .11	− .90
7	2.79	1.23	.05	− .97
8	2.72	1.19	.10	− .84
9	2.48	1.15	.38	− .67
10	2.38	1.12	.46	− .61
11	2.72	1.34	.17	− 1.22
12	2.84	1.32	− .01	− 1.21
13	2.76	1.41	.15	− 1.34
14	2.11	1.14	.80	− .24
15	2.30	1.19	.50	− .77
16	2.36	1.21	.47	− .82
17	2.14	1.13	.81	− .17
18	2.66	1.26	.17	− 1.02
19	2.60	1.21	.27	− .86
20	2.54	1.29	.32	− 1.02
21	2.79	1.25	.02	− 1.07
22	3.10	1.26	− .24	− .96
23	2.88	1.34	.04	− 1.17

*Note*. Likert scale is the following: 1 = “*Strongly Disagree*”, 2 = “*Disagree*”, 3 = “*Agree*”, 4 = “*Strongly Agree*”. *n* = 501

To initially investigate the factorial dimension, EFA was conducted with Varimax rotation and the extraction method of principal component analysis (PCA), in line with the original work (Gillispie et al. 2007a). The Kaiser–Meyer–Olkin (KMO) measure of sampling adequacy was 0.89 and Bartlett’s sphericity test was statistically significant (*χ*^*2*^= 6310.39, *df* = 253, *p* < 0.001).

The initial solution resulted in five components with eigenvalues > 1. However, an examination of the rotated matrix identified four items that had substantial factor loadings on more than one factor. These items reflected the themes of arousal (item 4 and item 6), over-involvement (item 10) and self-enhancement (item 15). To avoid ambiguity in the interpretation of the factors (Tabachnick et al. 1996), these items were removed. Furthermore, analyses revealed that item 9 (“*Just wanting to spend time with people who gamble*”), originally belonging to the *Over-Involvement* dimension, loaded substantially on the *Enjoyment/Arousal* factor and it was therefore moved into this factor. Thus, this item moved from a negative OE to a positive OE. This change can be legitimized by the fact that people may gamble as a way of spending time with friends and, compared to monetary expenditure, people are less likely to consider time spent on gambling as a negative consequence of gambling behaviour (see Kim et al. 2014).

A second EFA was performed on the 19 maintained items with Varimax rotation and the principal axis factoring (PAF) method of extraction. The solution (KMO = 0.87; Bartlett’s sphericity test: *χ*^*2*^= 4885.30, *df* = 171, *p* < 0.001) indicated a five-factor model, accounting for 60.77% of the overall variance (Table 2). Analysing the correlations among the five factors, we found significant, positive and strong correlations among the three positive outcome expectancy dimensions. The two negative outcome expectancy subscales were also significantly, positively and moderately correlated. Moreover, the *Over-Involvement* dimension was revealed to be significantly, positively and moderately correlated with *Enjoyment/Arousal* and *Money* and significantly, positively and strongly correlated with *Self-Enhancement*. Finally, a significant and positive low correlation emerged between *Emotional Impact* and *Self-Enhancement*, as well as a significant and negative low correlation between *Emotional Impact* and *Money*. A non-significant correlation was found between *Emotional Impact* and *Enjoyment/Arousal* (Table 2).

**Table 2:** Factor loadings of the items, eigenvalues and percentage of accounted variance for the five-factor solution of the nineteen items of the Gambling Expectancies Questionnaire – Modified (GEQ – MOD)

Item_a_	Item_b_	F1 *Enjoyment/* *Arousal*	F2 *Money*	F3 *Over-Involvement*	F4 *Emotional Impact*	F5 *Self-Enhancement*
1	1	.75				
2	2	.68				
3	3	.73				
5	4	.69				
7	5	.68				
8	6	.78				
9	7	.49				
21	17		.71			
22	18		.86			
23	19		.80			
11	8			.79		
12	9			.78		
13	10			.70		
18	14				.77	
19	15				.73	
20	16				.76	
14	11					.59
16	12					.59
17	13					.78
Eigenvalue		3.76	2.17	2.08	1.89	1.64
Accounted variance		19.77	11.43	10.96	9.97	8.64
		F1	F2	F3	F4	F5
F1		–				
F2		.49***	–			
F3		.35***	.29***	–		
F4		− .09	− .10*	.29***	–	
F5		.45***	.37***	.55***	.19***	–
M (*SD*)		29.78 (7.86)	8.77 (3.47)	10.70 (4.26)	7.81 (3.21)	8.91 (3.91)

*Note: Etraction method: Principal Axis Factoring; Rotation method: Varimax*

Item_a =_ Items are labelled following the order of the original *Gambling Expectancies Questionnaire*

Item_b =_ Items are labelled following the order of the *Gambling Expectancies Questionnaire – Modified*

*n* = 501

At this point, the five-factor structure was tested by CFA, employing the maximum likelihood (ML) method using AMOS 16 software (Arbuckle 2007). To verify the model’s fit, the following indices were taken into account: the comparative fit index (CFI; Bentler 1990), the Tucker-Lewis Index (TLI; Tucker et al. 1973) and the root mean square error of approximation (RMSEA; Steiger et al. 1980). For the TLI and CFI indices, values above 0.90 are indicative of acceptable fit, while values above 0.95 are indicative of excellent fit (Hu et al. 1999). The RMSEA value is considered acceptable when it is below 0.08 and good when it is below 0.05 (Kline 2010).

**Fig. 1:**
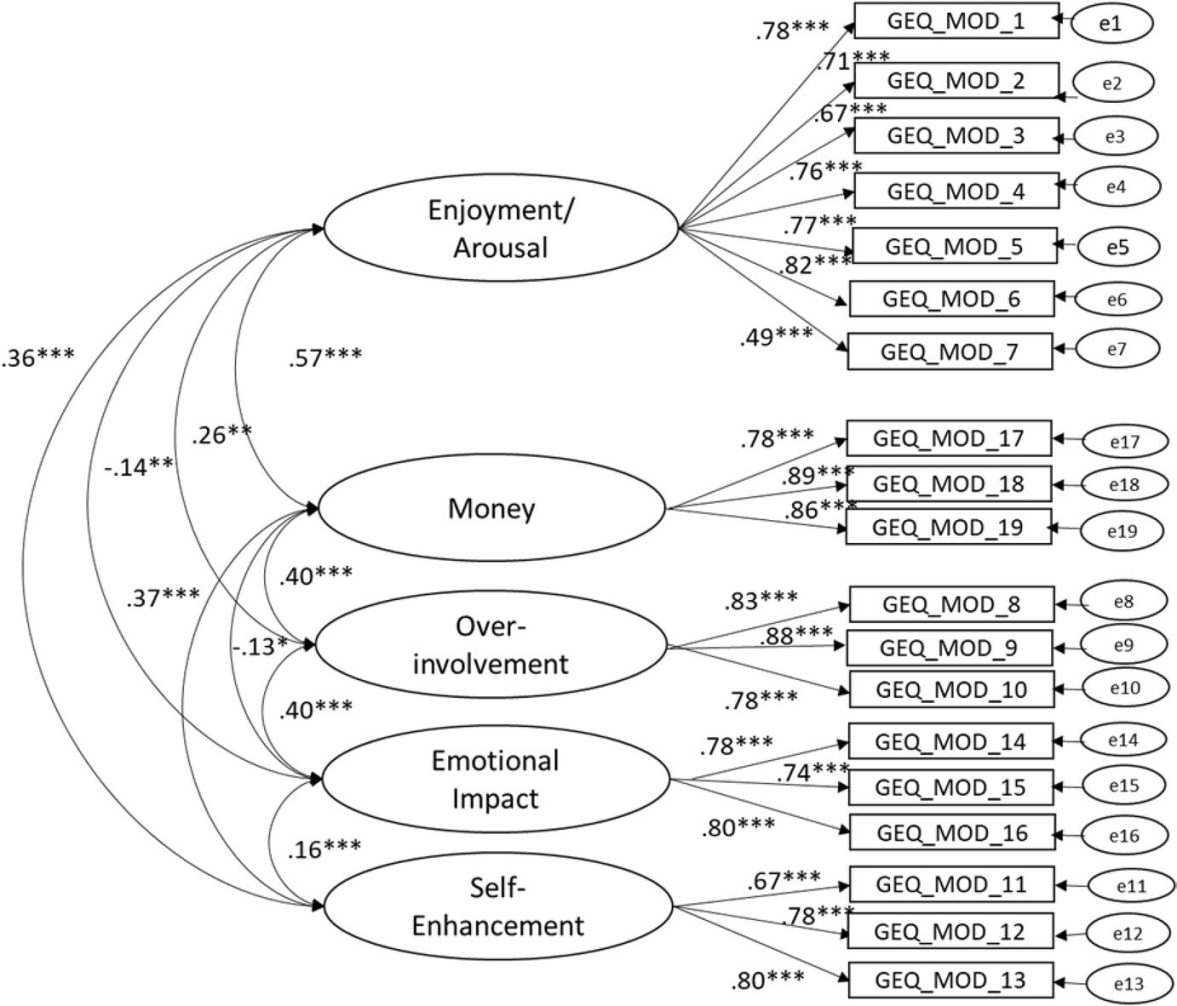
The five factor model of the GEQ – MOD. Note: standardized parameters, all significant at .001, n = 501

The results showed that the fit indices of the five-factor model were good (TLI = 0.928, CFI = 0.940, RMSEA = 0.063, 90% CI [0.056, 0.070]). All the factor loadings were significant at the 0.001 level and ranged from 0.49 to 0.89. Correlations among the five factors ranged from ± 0.12 and ± 0.58 (Fig. 1).

### Reliability and Validity

Cronbach’s alpha was 0.88 (95% CI [0.86, 0.90]) for the *Enjoyment/Arousal* subscale, 0.88 (95% CI [0.86, 0.90]) for the *Money* subscale, 0.85 (95% CI [0.83, 0.87]) for the *Over-Involvement* subscale, 0.81 (95% CI [0.78, 0.84]) for the *Emotional Impact* subscale and 0.79 (95% CI [0.76, 0.82]) for the *Self-Enhancement* subscale. All corrected item-total correlations were above 0.45. The McDonald’s Ω values were equivalent to the Cronbach’s alpha values (0.88, 0.85, 0.80, 0.81 and 0.88, respectively). Following the cut-offs proposed by the *European Federation of Psychologists’ Association* (EFPA; Evers et al. 2013), the internal consistency values were adequate for the *Self-Enhancement* subscale and good for the other subscales.

To analyse the criterion validity of the GEQ – MOD, we investigated its associations with gambling frequency. Gambling frequency was significantly and positively related with *Enjoyment/Arousal* (*r* = 0.28, *p* < 0.001) and *Money* (*r* = 0.26, *p* < 0.001) and negatively related with the *Emotional Impact* subscale (*r* = -0.15, *p* < 0.001). No significant correlations were found between gambling frequency and *Over-Involvement* (*p* = 0.637) or *Self-Enhancement* (*p* = 0.140). Subsequently, a multiple linear regression analysis was conducted to explain gambling frequency by OEs by introducing the GEQ – MOD dimensions related to gambling frequency as independent variables only. Results showed that the regression model was significant (*F* (3,492) = 19.37, *p* < 0.001) and explained 10% of the variance (*R*^*2*^ = 0.11, *AdjR*^*2*^ = 0.10). Significant predictors were revealed to be *Enjoyment/Arousal* (*ß* = 0.24, *p* < 0.001), *Money* (*ß* = 0.18, *p* < 0.001) and *Emotional Impact* (*ß* = -0.11, *p* = 0.029).

Concerning the relationship with gambling problem severity, we found that the GBS-A total score was significantly and positively related with *Enjoyment/Arousal* (*r* = 0.23, *p* < 0.001), *Money* (*r* = 0.26, *p* < 0.001), *Over-Involvement* (*r* = 0.19, *p* < 0.001) and *Self-Enhancement* (*r* = 0.19, *p* =  < 0.001). No significant correlations were found for *Emotional Impact* (*p* = 0.421). The subsequent multiple linear regression analysis (*F* (4,356) = 9.24, *p* < 0.001, *R*^*2*^ = 0.31, *AdjR*^*2*^ = 0.08) showed that *Enjoyment/Arousal* had a marginally significant predictive power (*ß* = 0.10, *p* = 0.094), while *Money* was a full significant predictor (*ß* = 0.18, *p* = 0.002) of GD symptoms.

## Discussion

This study was aimed at testing dimensionality, reliability and validity of the GEQ – MOD among adolescents. The findings supported the psychometric properties of the scale. Specifically, through an exploratory and confirmatory procedure, we obtained a 19-item instrument (GEQ – MOD) (see Appendix) with a five-factor structure, consistently with the GEQ (Gillespie et al. 2007a). The correlations among the subscales were in line with those found for the original instrument (Gillespie et al. 2007a). Concerning reliability, the five subscales of the GEQ – MOD showed good internal consistency values (Gillespie et al. 2007a).

As for validity, specific positive and negative OEs were found to be related to gambling frequency and gambling problem severity: *Enjoyment-Arousal* and *Money* positively predicted both frequency and GD symptoms, while *Self-Enhancement* only predicted gambling problem severity. *Emotional Impact* was a negative predictor of the frequency of gambling behaviour, whereas *Over-Involvement* was a positive predictor of gambling problem severity. Thus, results are overall consistent with Gillespie et al. (2007b), who reported that problem gamblers had higher scores in the *Over-Involvement* subscale with respect to at-risk and social gamblers. More generally, we confirmed that, although positive OEs are related to risky behaviours and negative OEs are negatively related with those behaviours (Colder et al. 2014), when explaining problem gambling in adolescence, the negative expectations played a role as positive predictors of problem gambling (Dowling et al. 2018; St-Pierre et al. 2014), consistent with the immediacy assumption theory (Gillespie et al. 2007b; Wickwire et al. 2010). The results also confirm that positive OEs are more influential determinants of addictive behaviours than negative OEs (Jones et al. 2001; Leigh et al. 2004).

## Study 2

After verifying the fundamental psychometric properties of dimensionality, reliability and validity, we aimed at testing measurement invariance of the GEQ – MOD across gambling status (non-gambler/gambler) and gender. This result would improve assessment of OEs related to gambling among all adolescents, independent of their actual gambling behaviour and also assessing equivalence among non-gamblers and gamblers. Thus, we sought to determine if the GEQ – MOD could assess GOEs among adolescent non-gamblers and gamblers. This finding would allow us to analyze GOEs even before the initiation of gambling behaviour. Concerning gender, the documented differences in gambling-related OEs have been found without previously assessing measurement invariance across genders of the instruments used to assess GOEs. Thus, any findings that address gender-related differences in youth must be interpreted with caution. After having tested measurement invariance, we aimed at investigating the differences across non-gamblers and gamblers as well as boys and girls.

## Methods

### Participants

The participants consisted of 1,894 Italian adolescents (61% males) between the ages of 14 and 19 (*M* = 15.68, *SD* = 0.71). The sample was recruited in high schools in Tuscany (17% vocational school, 40% technical school, 39% lyceum, 4% vocational training centres). Data collection occurred within the school prevention program *PRIZE – Prevenzione sui rischi correlati al gioco d’azzardo negli adolescenti – Prevention of gambling risk among adolescents*).^1^ Students received an information sheet that assured them that the data obtained would be handled confidentially and anonymously and they were asked to provide written informed consent. Parents of minors were required to provide consent on behalf of their children.

### Measures and Procedure

A socio-demographic questionnaire was first administered. To avoid a predetermined binary vision of adolescents' gender, we left open the question. The variable was made binary only in the data processing phase. Age in terms of years and months was also requested.

The *Gambling Expectancy Questionnaire – Modified* (GEQ – MOD) (see Appendix) was used to assess GOEs. The scale is composed of 19 items with a 5-point Likert scale from 1 (*totally disagree*) to 5 (*totally agree*). Seven items measure *Enjoyment/Arousal*, three items assess *Self-Enhancement* and three items are indicative of *Money*. As for the remaining items, three measure *Over-Involvement* and three *Emotional Impact*.

To investigate gambling behavior status, we administered the first section of the GBS-A (Primi et al. 2015). As reported in Study 1, this section investigates gambling frequency (never, sometimes in the year, sometimes in the month, sometimes in the week, daily) of participation during the last year for ten gambling activities (card games, bets on games of personal skill, bets on sports games, bets on horse races, bingo, slot machines, scratch cards, lotteries, online games and private bets with friends). Based on their responses, participants were classified as non-gamblers (no gambling behaviour) or gamblers (gambling on at least one activity; Welte et al. 2009).

The scales were administered individually during class time, in the following order: GEQ – MOD and then the first section of the GBS-A. Students were provided with a brief introduction to the study and with instructions. Answers were collected in a paper-and-pencil format and data collection was completed in about 20 min and then entered into a database for analyses.

## Results

Missing data analysis revealed that 10 cases had more than 10% of missing items (Kline 2010) at the GEQ – MOD and the first section of the GBS-A. These cases were subsequently removed. Moreover, in the GEQ – MOD there were other 3 cases with more than 10% of missing data, leading to a data sample size of 1,881 adolescents. On the first section of the GBS – there were 3 cases exceeding 10% of missing data and these were excluded for the analyses concerning invariance across non-gamblers and gamblers.

### GEQ – MOD Invariance Across Non-Gamblers and Gamblers

Measurement invariance across non-gamblers (n = 460) and gamblers (n = 1,418) was also analysed. The five-factor solution was confirmed in both groups, with good fit indices among non-gamblers (CFI = 0.94; TLI = 0.93; RMSEA = 0.066, 90% CI [0.059, 0.073]) and with standardized factor loadings significant at the 0.001 level, ranging from 0.63 to 0.91. For gamblers, the model similarly reached adequate fit indices (CFI = 0.93; TLI = 0.92; RMSEA = 0.063, 90% CI [0.059, 0.067]). Standardized factor loadings ranged from 0.55 to 0.87 and were all significant at the 0.001 level.

Analyses were then conducted by performing hierarchical nested CFA. First, the default independence model was fitted (*χ*^2^ = 16,610.55, *df* = 380, *p* < 0.001) and the configural, the metric factorial, the scalar, the structural variance and covariances and the measurement error invariances were verified, as the ∆CFI and the ∆RMSEA criteria were satisfied (Table 3).

**Table 3:** Fit statistics of the GEQ—MOD for each level of structural and measurement invariance across gambling behavior status

Model	*χ*^2^(*df*)	*χ*^2^/*df*	*p*	CFI	RMSEA [CI 90%]	Model Comparison	∆*χ*^2^	∆*df*	*p*	∆CFI	∆RMSEA
1. Invariance of model configuration	1373.49 (284)	4.84	< .001	.933	.045 [.043–.048]	–	–	–	–	–	
2. Invariance of factor loading	1393.69 (298)	4.68	< .001	.932	.044 [.042–.047]	Model 1–Model 2	20.20	14	.124	.001	.001
3. Invariance of intercepts	1512.33 (317)	4.77	< .001	.926	.045 [.043–.047]	Model 2–Model 3	118.64	19	.000	.006	.001
4. Invariance of structural variances/covariances	1542.63 (332)	4.65	< .001	.925	.044 [.042–.046]	Model 3–Model 4	30.30	15	.011	.001	.001
5. Invariance of measurement error variances/covariances	1617.68 (351)	4.61	< .001	.922	.044 [.042–.046]	Model 4–Model 5	75.05	19	.000	.003	.000

*Notes*: *χ*2 = chi square test; *df* = degrees of freedom; CFI = comparative fit index; RMSEA = root mean square error of approximation; CI = confidence interval; ∆*χ*^2^ = Satorra–Bentler scaled difference; ∆*df* = difference in degrees of freedom between nested models; *p* = probability value of ∆*χ*^2^ test; ∆CFI = difference between robust CFIs of nested models, ∆RMSEA = difference between robust RMSEAs of nested models. *n* = 1878

### GEQ – MOD Invariance Across Boys and Girls

Measurement invariance across gender was also investigated. As a prerequisite, we tested the final five-factor model separately for males and females (Byrne 2004) using AMOS 16 (Arbuckle 2007). As 47 participants did not report their gender, the analyses were run with 1,844 cases (1,116 males and 728 females). The model showed good fit indices among boys (CFI = 0.94; TLI = 0.93; RMSEA = 0.062, 90% CI [0.057, 0.066]), with standardized factor loadings significant at the 0.001 level and ranging from 0.57 to 0.88. For girls, the model also reached acceptable fit indices (CFI = 0.91; TLI = 0.89; RMSEA = 0.076, 90% CI [0.070, 0.081]). Standardized factor loadings ranged from 0.58 to 0.87 and were all significant at the 0.001 level.

Analyses were subsequently conducted by performing hierarchical nested confirmatory factor analyses; invariance was evaluated not only with ∆*χ*^2^, which is sensitive to sample size (Chen 2007), but also using the criterion of ∆CFI < 0.01 (Chen 2007; Cheung et al. 2002) (that is, the CFI value for a more restrictive model should not be more than 0.01 below the preceding, less restrictive, model) and the equivalent cut-off of 0.015 for RMSEA.

First, the default independence model was fitted (*χ*^2^ = 16,465.22, *df* = 380, *p* < 0.001). As reported in Table 4, in addition to configural invariance, metric factorial invariance was supported, confirming that the factor loadings were equal across genders. The scalar invariance, which constrained intercepts to be invariant across groups, was tested and subsequently, the equivalence of structure variances and covariances was also tested, along with the invariance of measurement error. Hence, the testing of the equality of the items’ variance and covariances met the ∆CFI and the ∆RMSEA criterion.

**Table 4:** Fit statistics of the GEQ—MOD for each level of structural and measurement invariance across gender

Model	*χ*^2^(*df*)	*χ*^2^/*df*	*p*	CFI	RMSEA [CI 90%]	Model Comparison	∆*χ*^2^	∆*df*	*p*	∆CFI	∆RMSEA
1. Invariance of model configuration	1471.98 (284)	5.18	< .001	.926	.047 [.045–.050]	–	–	–	–	–	–
2. Invariance of factor loadings	1479.03 (298)	4.96	< .001	.927	.046 [.044–.049]	Model 1–Model 2	7.05	14	.933	.001	.001
3. Invariance of intercepts	1627.75 (317)	5.13	< .001	.919	.047 [.045–.050]	Model 2–Model 3	148.75	19	.000	.008	.001
4. Invariance of structural variances/covariances	1663.30 (332)	5.01	< .001	.917	.047 [.044–.049]	Model 3–Model 4	35.55	15	.002	.002	.000
5. Invariance of measurement error variances/covariances	1700.16 (351)	4.84	< .001	.916	.046 [.043–.048]	Model 4–Model 5	36.86	19	.008	.001	.001

*Notes*: *χ*^2^ = chi square test; *df* = degrees of freedom; CFI = comparative fit index; RMSEA = root mean square error of approximation; CI = confidence interval; ∆*χ*^2^ = Satorra–Bentler scaled difference; ∆*df* = difference in degrees of freedom between nested models; *p* = probability value of ∆*χ*^2^ test; ∆CFI = difference between robust CFIs of nested models, ∆RMSEA = difference between robust RMSEAs of nested models. *n* = 1,844 cases

### Comparisons Across Gambling Behaviour Status and Genders

Regarding gambling behaviour status, gamblers had higher positive OEs than non-gamblers in the *Enjoyment/Arousal* and *Money* dimensions and these differences were characterized by moderate effect sizes. Concerning gender, the results showed significant and moderate-in-size differences in the *Over-Involvement* and *Emotional Impact* subscales, indicating that female adolescents had higher negative outcome expectations related to gambling than male adolescents (Table 5).

**Table 5:** Means comparisons across gender and gambling frequency levels for the GEQ – MOD dimensions

GEQ – MOD dimensions	Non-gamblers (*n* = 460)	Gamblers (*n* = 1418)	*t* *(1876)*	*p*	Cohen’s *d*
	M (SD)	M (SD)			
*Enjoyment/Arousal*	16.30 (6.01)	18.61 (5.74)	− 7.42	< .001	.39
*Money*	8.33 (3.66)	9.33 (3.42)	− 5.37	< .001	.28
*Over-Involvement*	8.43 (3.77)	8.29 (3.63)	.75	.451	.04
*Emotional Impact*	6.84 (2.97)	6.80 (2.91)	.25	.805	.01
*Self-Enhancement*	6.22 (3.14)	6.39 (3.05)	− 1.07	.287	.05

GEQ – MOD dimensions	Male Adolescents (*n* = 1116)	Female adolescents (*n* = 728)	*t* *(1842)*	*p*	Cohen’s *d*
	M (SD)	M (SD)			
*Enjoyment/Arousal*	18.38 (6.03)	17.61 (5.62)	2.77	.006	.13
*Money*	9.15 (3.55)	9.05 (3.42)	.65	.515	.03
*Over-Involvement*	7.89 (3.58)	8.98 (3.73)	− 6.28	< .001	.30
*Emotional Impact*	6.70 (2.97)	6.98 (2.85)	-2.04	.041	.10
*Self-Enhancement*	6.01 (2.92)	6.85 (3.23)	− 5.85	< .001	.27

## Discussion

The aim of this study was to test the measurement invariance of the GEQ – MOD across gambling behaviour status and gender and to conduct fair comparisons about GOEs between adolescent non-gamblers and gamblers as well as among male and female adolescents. The current results supported invariance of the GEQ – MOD across gambling behaviour and gender in adolescence, assuring that valid comparisons between non-gamblers and gamblers as well as boys and girls can be conducted among adolescents. These results are relevant because, to date, there has been no evidence concerning the invariance of instruments to measure GOEs in youth. Detecting invariance of the GEQ – MOD across gambling status and gender is important to better understand how OEs affect gambling involvement and how gender characteristics shape the development and maintenance of OEs, while also assessing – via fair and unbiased comparisons – differences among adolescents concerning gambling participation and gender.

As for gambling behaviour status, gamblers displayed higher levels of *Enjoyment/Arousal* (Brown 1986; Coventry et al. 1997) and *Money*, consistent with the fact that expecting to gain money from gambling characterized adolescents who gamble (Delfabbro et al. 2003). However, no differences were found concerning the other subscales, confirming the socio-cultural nature of GOEs, that can be present also before the first gambling experience (e.g., Gillespie et al. 2007b; Kuntsche et al. 2007). Concerning gender, boys and girls showed differences in terms of negative OEs (Gillespie et al. 2007b), specifically on *Over-Involvement* and *Emotional Impact*, with females having higher NOEs than males. Concerning the positive OEs, while boys had higher scores on *Enjoyment/Arousal*, female adolescents had higher scores than male adolescents on *Self-Enhancement*. This is an interesting result that may explain the increasing female gambling participation, although the gender ratio between male and female adolescents in the prevalence of problem gamblers still exists (Calado et al. 2017).

## Conclusion

GOEs have been found to be among the predictors of gambling behaviour in adolescents (e.g. Dowling et al. 2018; Gillispie et al. 2007b; Wickwire et al. 2016, 2010). Thus, having adequate and effective measurement tools to assess GOEs in youth is fundamental. Nonetheless, there is a paucity of instruments assessing this construct among adolescents (Gillespie et al. 2007a; Wickwire et al. 2010; Wong et al. 2012). Among such instruments, the GEQ (Gillespie et al. 2007a) is a promising tool because of its brevity and construct validity. However, it cannot be legitimately administered to adolescents who have never had gambling experiences, making it difficult to use it for early prevention purposes. To make the GEQ a more useful preventative instrument, this study aimed to modify it so that it would be adequate to be administered to all adolescents.

The current work attests that the GEQ – MOD is a reliable and valid multidimensional self-report instrument to measure positive and negative OEs among adolescents. Importantly, this tool has found to be invariant across gambling behaviour status and gender, i.e., it is able to equivalently assess GOEs among non-gamblers and gamblers as well as male and female adolescents. Adolescents who gamble are more prone to expect enjoyment/arousal and money from gambling with respect to adolescents who do not gamble. Any difference emerged concerning *Self-Enhancement* and the negative OEs. Concerning gender, while boys tend to have higher OEs than girls with respect to *Enjoyment/Arousal*, girls revealed to be more likely than boys to endorse items related to *Self-Enhancement*. As for the negative OEs, girls have been found to have higher levels than boys.

Such an instrument among adolescents represents a useful tool for research and practice. Indeed, given the complexity and multidimensionality of the construct of OEs, it is fundamental to have tools capable of assessing different kinds of expectancies, of identifying the most relevant and problematic expectancies and ascertaining which need interventions. To that end, the GEQ – MOD appears to be particularly useful. From a practical perspective, the scale appears to be appropriate for large, multivariate studies in which many tests and scales need to be administered. For research purposes, it would be interesting to employ the GEQ – MOD to investigate the role of GOEs in explaining gambling behaviour in youth by deepening its role as a mediator in the relationship between risk factors and behavioural outcomes as the literature about risky behaviours suggests (e.g. Colder et al. 2014; Settles et al. 2014). Moreover, it would be interesting to develop a better understanding of the reciprocal association of OEs with cognitive distortions related to gambling, which seem to be erroneous and misinterpreted expectancies (van Holst et al. 2012), perhaps due to the excessive gambling behaviour. It is important to test whether having specific positive OEs related to gambling may represent a risk factor for the development of erroneous cognitions once an adolescent begins to gamble with a considerable frequency. In terms of practice, the GEQ – MOD should be adopted to select adolescents with high levels of positive OEs in order to address targeted expected consequences from gambling. Moreover, the GEQ – MOD could be used to verify the effectiveness of interventions addressed to strengthen negative OEs. More broadly speaking, the GEQ- MOD makes it possible to assess the efficacy of educational programs aiming to modify adolescents’ OEs related to gambling. In this regard, it is important to underly that the influence of perceived expected benefits on gambling is strictly linked to gambling advertisement (see Binde et al. 2019; Parke et al. 2015, for reviews). Indeed, gambling is typically advertised as a harmless form of entertainment and an enjoyable fun, leisure time activity (e.g., Derevensky et al. 2010; Pitt et al. 2016), while the harmful consequences of excessive gambling are generally framed as an issue of choice (Korn et al. 2003). Thus, the underlying perceived message is that winning is easy, the chance of winning is high and gambling is an easy way to acquire money and wealth. Young people are exposed to such kind of messages, through pop-up ads on the Internet, newspapers, radio and TV, magazines. Research suggest that there is a proportion of adolescents who gamble because of these messages and that boys, older youth and problem gamblers are the most susceptible to the negative effects of advertisements (Derevensky et al. 2010) in terms of attitudes toward gambling. For these reasons, preventive interventions aimed at modifying GOEs must address environmental determinants. Additionally, given the culturally-based nature of expectancies in general (Friedman et al. 2006; Peele et al. 2000) and specifically in the gambling domain (Gillispie et al. 2007; Wickwire et al. 2010; Wong et al. 2012), it is important for future research to investigate which positive and negative expectancies adolescents perceive towards gambling among different cultural groups. The employment of the GEQ – MOD could be a valid instrument to be used.

Despite some strengths, as the conduction of two studies and the involvement of a large sample size in Study 2, the results must be read with certain limitations in mind. Future studies should include larger national samples covering a greater age range in order to analyze the measurement invariance of the tool also across age. Finally, it would be useful to investigate the convergent and divergent validity of the GEQ – MOD.

The current work suggests that the GEQ – MOD is a reliable and valid multidimensional self-report instrument to assess positive and negative OEs among adolescents and will provide clinicians, prevention specialists and public health officials with useful information.

## Appendix

### The GEQ – MOD items in the English version.

Here below you will find a series of statements. For each of them indicate how much you agree making a cross on the answer you choose. Remember that:

1 = totally disagree

2 = quite disagree

3 = neither agree nor disagree

4 = quite agree

5 = totally agree

If you were gambling, gambling would make you….

**Table:**

ENJOYMENT-AROUSAL
1. Having fun
2. Feeling more relaxed
3. Stopping being bored
4. Spending time with people I like
5. Enjoying myself
6. Having a good time
OVER-INVOLVEMENT
7. Wanting to spend time with people who gamble
8. Wanting to gamble more and more
9. Getting hooked
10. Being not able to stop
SELF-ENHANCEMENT
11. Having friends and classmates who think I’m cool
12. Feeling in control
13. Feeling more accepted by people
EMOTIONAL IMPACT
14. Feeling guilty
15. Feeling in over my head
16. Feeling ashamed of myself
MONEY
17. Making a profit
18. Winning money
19. Getting rich

## Footnotes

1 This program is one of the initiatives included in the Tuscany’s plan to prevent problem gambling (Resolution of the Tuscany Region n. 771, 9 July 2018).
